# Effects of different doses and serotypes of LPS on blood-brain barrier permeability in Sprague-Dawley rats

**DOI:** 10.1186/cc11959

**Published:** 2013-03-19

**Authors:** E Senturk, F Esen, P Ergin Ozcan, G Orhun, N Orhan, N Arican, M Kucuk, M Kaya

**Affiliations:** 1 University of Istanbul, Turkey

## Introduction

The blood-brain barrier (BBB) is highly restrictive of the transport of substances between blood and the central nervous system. Lipopolysaccharide (LPS) from Gram-negative bacteria was reported to affect the permeability of the BBB. BBB disruption using a LPS is used as a model of septic encephalopathy in mice. The present study was designed to investigate the effects of different doses and serotypes of LPS on BBB integrity in Sprague-Dawley rats.

## Methods

Male Sprague-Dawley rats weighing 200 to 250 g were used in the study. Rats were given two different types of LPS (026:B6-L5543 and 026:B6-L2762) in different doses (3, 5, and 103mg/kg; i.v.). Rectal temperature and arterial blood pressure measurements were recorded for sepsis severity. The changes in the BBB permeability were measured using the Evans blue (EB) and sodium fluorescein (NaFl) dye extravasation techniques 24 hours after LPS administration.

## Results

Both LPS serotypes showed comparable arterial blood pressure and rectal temperature recordings and the severity of the disease increased with the increasing doses (5 mg/kg and 10 mg/kg) of LPS and the mortality rates were found to be 29% and 63% respectively. The extravasated contents of EB and NaFl tracers did not significantly increase in brain parenchyma following the administration of different doses of LPS with different serotypes.

## Conclusion

Our results showed no disruption to BBB by two different serotypes of LPS even administered in increasing doses. These result indicate that the BBB integrity of Sprague-Dawley rats are resistant to the effects of two different serotypes of LPS.

